# Identifying similar transcripts in a related organism from de Bruijn graphs of RNA-Seq data, with applications to the study of salt and waterlogging tolerance in *Melilotus*

**DOI:** 10.1186/s12864-019-5702-5

**Published:** 2019-06-06

**Authors:** Shuhua Fu, Peter L. Chang, Maren L. Friesen, Natasha L. Teakle, Aaron M. Tarone, Sing-Hoi Sze

**Affiliations:** 1 Department of Biochemistry & Biophysics, Texas A&M University, College Station, 77843 TX USA; 2 Molecular and Computational Biology Section, Department of Biological Sciences, University of Southern California, Los Angeles, 90089 CA USA; 3 Department of Crop and Soil Sciences, Washington State University, Pullman, 99164 WA USA; 4 Department of Plant Pathology, Washington State University, Pullman, 99164 WA USA; 5 Centre for Ecohydrology, The University of Western Australia, 35 Stirling Highway, Crawley, 6009 WA Australia; 6 School of Plant Biology (M084), Faculty of Natural and Agricultural Sciences, The University of Western Australia, 35 Stirling Highway, Crawley, 6009 WA Australia; 7 Department of Entomology, Texas A&M University, College Station, 77843 TX USA; 8 Department of Computer Science and Engineering, Texas A&M University, College Station, 77843 TX USA

**Keywords:** de Bruijn graph, RNA-Seq, *Melilotus*

## Abstract

**Background:**

A popular strategy to study alternative splicing in non-model organisms starts from sequencing the entire transcriptome, then assembling the reads by using de novo transcriptome assembly algorithms to obtain predicted transcripts. A similarity search algorithm is then applied to a related organism to infer possible function of these predicted transcripts. While some of these predictions may be inaccurate and transcripts with low coverage are often missed, we observe that it is possible to obtain a more complete set of transcripts to facilitate possible functional assignments by starting the search from the intermediate de Bruijn graph that contains all branching possibilities.

**Results:**

We develop an algorithm to extract similar transcripts in a related organism by starting the search from the de Bruijn graph that represents the transcriptome instead of from predicted transcripts. We show that our algorithm is able to recover more similar transcripts than existing algorithms, with large improvements in obtaining longer transcripts and a finer resolution of isoforms. We apply our algorithm to study salt and waterlogging tolerance in two *Melilotus* species by constructing new RNA-Seq libraries.

**Conclusions:**

We have developed an algorithm to identify paths in the de Bruijn graph that correspond to similar transcripts in a related organism directly. Our strategy bypasses the transcript prediction step in RNA-Seq data and makes use of support from evolutionary information.

**Electronic supplementary material:**

The online version of this article (10.1186/s12864-019-5702-5) contains supplementary material, which is available to authorized users.

## Background

As the advance in high-throughput sequencing enables the generation of large volumes of genomic information, it provides researchers the opportunity to study non-model organisms even in the absence of a fully sequenced genome. These studies often start from sequencing the entire transcriptome, while additional software is applied to process the data. An important mechanism to study is alternative splicing, which is crucial to a variety of biological functions. The goal of these studies is to recover as many isoforms as possible in order to understand the underlying biological processes.

In the presence of a reference database, there are two strategies for analyzing transcriptome data. Mapping-first algorithms perform splice-aware alignment of the reads to the reference genome to reconstruct the transcripts [1, 2]. While these algorithms can construct transcripts independent of known splice sites and identify novel mRNA products, they only allow very few differences during the alignment. Alternatively, when a reference genome is not available but a reference transcriptome is available, transcript quantification algorithms can be applied to analyze differential expression of genes [3, 4].

In the absence of a reference database, an alternative strategy is to employ de novo sequence assembly algorithms [5–12]. A popular strategy of transcriptome assembly algorithms is to assemble the reads by obtaining a de Bruijn graph that represents the transcriptome [12–15].

Although the de Bruijn graph contains all branching possibilities, an additional step is needed to obtain predicted transcripts from the graph. To obtain information about possible function of these predicted transcripts, a similarity search algorithm such as BLAST [16] is then applied to identify similar transcripts in a related organism. In non-model organisms where a fully sequenced genome is not available, this step is the most reliable way to facilitate possible functional assignments. Since the predicted transcripts are constructed based on coverage information, one shortcoming of this approach is that sequences with low coverage are often ignored leading to missed transcripts. The later BLAST step to a related organism then starts from this relatively incomplete set of predicted transcripts.

**Fig. 1:**
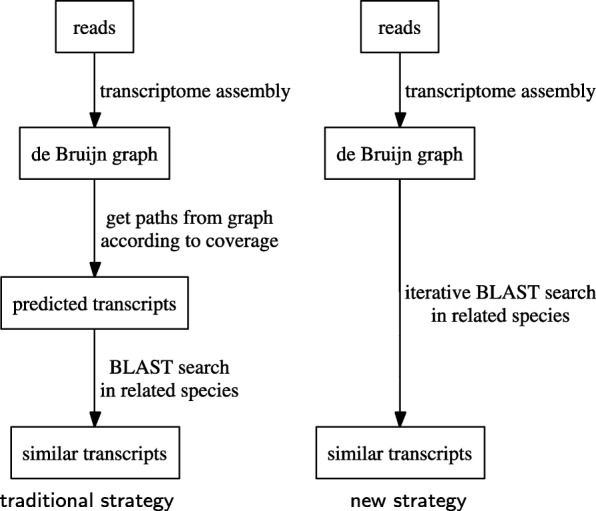
Difference between traditional strategy to obtain similar transcripts and our new strategy that bypasses the transcript prediction step

Instead of performing similarity search from the predicted transcripts, we observe that it is possible to obtain a more complete set of similar transcripts if we start the search from the de Bruijn graph directly (see Fig. 1). This strategy bypasses the transcript prediction step and makes use of support from evolutionary information. Since the graph retains more information from the transcriptome data, transcripts that have low coverage can still be recovered if they have high similarity to the ones from the related organism. Wu et al. [17] employed a similar strategy in metagenomics to extract paths directly from the de Bruijn graph that correspond to homologous genes from closely related species. Bao et al. [18] utilized genomic information from the same organism or a related organism (instead of transcripts from a related organism) to improve de novo transcriptome assemblies by first identifying exons from alignments.

While the strategy of applying BLAST from each node in a de Bruijn graph to a related organism can already give a lot of hits, it is possible that some significant hits are missed since the sequence within a node may be too short. There is a need to identify paths in the de Bruijn graph that are similar to transcripts from the related organism. Since the number of possible paths that can be constructed from the de Bruijn graph can be very large, it is not feasible to enumerate all of them.

We develop a heuristic extension algorithm that starts by enumerating short paths in the de Bruijn graph, and iteratively extends these paths in the most promising direction rather than in all possible directions. This procedure generalizes the BLAST algorithm to allow a non-linear query structure instead of a query sequence. Fu et al. [19] utilized a similar heuristic algorithm to simultaneously extend paths in two de Bruijn graphs in order to compare the transcriptomes of two related organisms at the same time. Zhong et al. [20] employed a gene-centric approach in metagenomics to extend an assembly graph structure by identifying reads that are related to assembled protein sequences. Note that our strategy is different from the one in [17] that uses optimal alignment to extend paths due to the smaller scale of metagenomic data. We compare the performance of our algorithm that starts the search from the de Bruijn graph against existing algorithms that employ the strategy of first obtaining predicted transcripts then applying BLAST to obtain similar transcripts.

We validate our algorithm by extracting reads from publicly available RNA-Seq libraries. We construct new RNA-Seq libraries for the non-model organisms *Melilotus albus* and *Melilotus siculus*, and apply our algorithm to study salt and waterlogging tolerance in these two species.

## Methods

Given a set of reads and a parameter *k*, a popular strategy of transcriptome assembly algorithms is to assemble these reads into a de Bruijn graph that represents the transcriptome. By taking each *k*-mer that appears within the reads as a vertex, and connecting two *k*-mers by a directed edge if the (*k*−1)-suffix of the first *k*-mer is the same as the (*k*−1)-prefix of the second *k*-mer, the de Bruijn graph implicitly assembles the reads by linking together the same *k*-mer that comes from different reads [21, 22]. This strategy is very popular among short read assembly algorithms [6, 7, 9–11].

To minimize the effect of sequencing errors, these algorithms remove short tips and further simplify the de Bruijn graph by collapsing similar paths. Each linear path that contains a sequence of vertices with no branches is collapsed into a single node, and a *k*-mer coverage cutoff *c* is imposed to remove low coverage nodes [9–11]. We develop an algorithm to identify paths in the de Bruijn graph that correspond to similar transcripts in a related organism. Each extracted path can be considered as a predicted transcript in the original organism.

### Initial choice of contigs to extend

For each transcript in a related organism, our goal is to recover the best path in the de Bruijn graph that corresponds to the transcript. Our approach is based on the seed-extension strategy that starts from short paths, and iteratively extends these paths in the most promising direction. We start the search from nodes in a de Bruijn graph that correspond to contigs from short read assembly algorithms [9–11].

Given a de Bruijn graph, a database of known transcripts in a related organism and an *e*-value cutoff *e*_*f*_, we first apply BLAST from each node in the de Bruijn graph to the transcript database to obtain all hits with *e*-value below *e*_*i*_, where *e*_*i*_>*e*_*f*_. The extra *e*-value cutoff *e*_*i*_ is chosen to allow the initial seed nodes to be of lower quality. Some of these nodes can be extended later into longer paths that are of higher quality.

For each transcript in the database, we extract the top *n* nodes in the de Bruijn graph that give the best BLAST hits to it, where *n* is a given parameter. The resulting collection of nodes over all transcripts in the database becomes the set of all nodes that our heuristic extension algorithm will start from, which are the ones that are most likely to have correspondences with transcripts in the database. Note that more stringent values of *k* and the *k*-mer coverage cutoff *c* can provide longer nodes to start with but can also lead to missed nodes.

### Heuristic extension

**Fig. 2:**
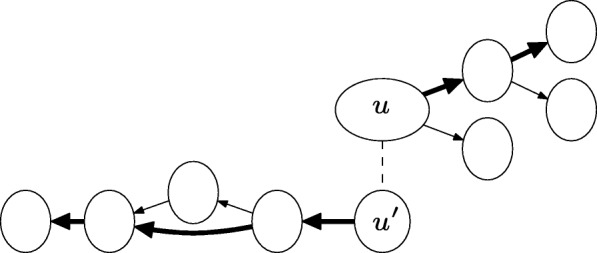
Illustration of the heuristic extension procedure from a node *u* and its twin node *u*^′^ until the *e*-value no longer improves (extension proceeds along bold edges)

For each node *u* in the collection, we extend its sequence by one node along all outgoing edges from *u*, and apply BLAST from each of these extended sequences to the transcript database (see Fig. 2). If at least one of these extended sequences gives a better *e*-value, we extract the top extended path that gives the best *e*-value. We repeat the extension procedure starting from this new path until either there are no more outgoing edges to extend from or the *e*-value no longer improves.

Note that during each extension, only one best direction is chosen. Extending in more than one direction is very time-consuming since the number of possibilities can be exponential even in the absence of cycles. Although it is possible that the real best path may be missed, it is still possible to resolve different isoforms since the heuristic extension procedure starts independently from multiple nodes, some of which may be specific to particular isoforms. The procedure can be applied even in the presence of cycles in the de Bruijn graph since the *e*-value cannot improve indefinitely.

We perform a similar procedure on the node *u*^′^ that is the twin node of *u*, which represents the reverse complementary sequence of *k*-mers on the opposite strand, and try to extend it in the opposite direction (see Fig. 2). In addition to adding these two extended paths from *u* and *u*^′^ to the set of candidate paths, we also merge the twin path that is complementary to the extended path from *u*^′^ with the extended path from *u* to obtain a longer path. We add the merged path to the set of candidate paths and identify its best BLAST hit in the transcript database.

### Extraction of similar transcripts

At the end of the procedure, for each transcript in the database, we report the top path that gives the best *e*-value to it among all the candidate paths if such a path exists, where the set of candidate paths includes all paths that BLAST has been applied. Only the nodes of a path that are in the best BLAST alignment are reported. It is possible that some of these paths may be the same or very similar for different transcripts in the database.

### *Melilotus* RNA-Seq

mRNA was extracted from *Melilotus albus* and *Melilotus siculus* using a Qiagen Oligotex mRNA mini kit. Fragmentation of mRNA was done using an Ambion fragmentation buffer. Construction of the cDNA library was based on the Illumina protocol. First strand cDNA synthesis was done using Random Hexamer Primers (Invitrogen) and second strand synthesized using a DNA Polymerase 1 (Promega). End repair was carried out to create uniform blunt ends (Epicentre End-IT repair kit). Unique 4 bp adaptors (Illumina) were added so that the libraries could be pooled for sequencing. An ‘A’ base was added using a Klenow enzyme (3 ^′^ to 5 ^′^ exo minus, NEB) and adaptor ligation was performed using Epicentre Fast-Link DNA ligation kit. The cDNA template was run on a 2% agarose gel at 120 V for 60 minutes and fragments of approximately 200–500 bp were removed and purified (Zymo gel purification kit). The purified cDNA template was PCR enriched using the Illumina primers and a Phusion polymerase (NEB). The library was quantified using an Invitrogen Qubit fluorometer. Libraries were sequenced on an Illumina Genome Analyzer II under normal conditions and conditions associated with salt tolerance and/or waterlogging tolerance as single-end 100 bp reads, which were trimmed to 71 bp.

## Results and discussion

To assess the performance of our algorithm extContig, we extracted reads from publicly available RNA-Seq libraries (see Table 1). We validate our algorithm on model organisms by applying BLAST to a database of annotated transcripts in each model organism itself and in two other related model organisms with varying evolutionary distances, including *Schizosaccharomyces pombe* against another yeast species *Saccharomyces cerevisiae* and another fungus *Neurospora crassa*, *Drosophila melanogaster* against another *Drosophila* species *Drosophila pseudoobscura* and mosquito *Anopheles gambiae*, *Homo sapiens* against squirrel monkey *Saimiri boliviensis* and mouse *Mus musculus*, and *Arabidopsis thaliana* against another *Arabidopsis* species *Arabidopsis lyrata* and rice *Oryza sativa*.

**Table 1:** Data sets used in the evaluation of our heuristic extension algorithm, with organism indicating the starting organism, related organisms indicating the related model organisms that BLAST is applied to, library indicating the total number of libraries, size indicating the total number of bases in all the reads after quality trimming, and reference indicating the publication that describes the libraries

Organism	Related organisms	Library	Size	Reference
*S. pombe*	*S. cerevisiae*	32	17 G	[12]
	*N. crassa*			
*D. melanogaster*	*D. pseudoobscura*	13	9.6 G	[37]
	*A. gambiae*			
*H. sapiens*	*S. boliviensis*	4	16 G	[38]
	*M. musculus*			
*A. thaliana*	*A. lyrata*	5	16 G	[39]
	*O. sativa*			
*L. sericata*	*D. melanogaster*	9	4.6 G	[23]
*H. glaber*	*H. sapiens*	13	61 G	[24]
*C. sociabilis*	*H. sapiens*	10	66 G	[25]
*C. arietinum*	*A. thaliana*	3	8.6 G	[26]
*M. albus*	*A. thaliana*	12	5.5 G	New data
*M. siculus*	*A. thaliana*	12	5.4 G	New data

We evaluate the performance of our algorithm on publicly available RNA-Seq libraries from four non-model organisms. The blow fly *Lucilia sericata* is important in medicine, forensic science and agriculture due to its filth feeding habits, its use in maggot therapy, its colonization of human and animal remains, and its ability to cause myiasis in vertebrates [23]. The naked mole rat *Heterocephalus glaber* is important in medicine and in biomedical research due to its resistance to cancer and delayed aging, and its ability to live in adverse conditions [24]. The rodent *Ctenomys sociabilis* is important in the study of social behavior of mammals and the relationship to gene expression [25]. The chickpea *Cicer arietinum* is one of the most consumed legume crops that grows in arid areas with low productivity [26]. Similarity search is performed from *L. sericata* to the model organism *D. melanogaster*, from *H. glaber* and *C. sociabilis* to the model organism *H. sapiens*, and from *C. arietinum* to the model organism *A. thaliana*. The searches that are applied against the same model organism have varying evolutionary distances.

We have constructed new RNA-Seq libraries for the non-model organisms *Melilotus albus* and *Melilotus siculus*, which are important in the study of salt and waterlogging tolerance of forage plants [27]. Genomic information on the species will enable the dissection of coumarin production that can be utilized in pharmaceutical development [28]. Similarity search is performed from *M. albus* and *M. siculus* to the model organism *A. thaliana*.

We trimmed each read by removing all positions including and to the right of the first position that has a quality score of less than 15. For smaller data sets (including *D. melanogaster*, *L. sericata*, *C. arietinum*, *M. albus* and *M. siculus*), we compare the performance of our heuristic extension algorithm extVelvet starting from the de Bruijn graph given by Velvet [9] against the performance of Oases [14] that is a postprocessing module of Velvet. Since Oases requires that Velvet is run without coverage cutoff and then applies the coverage cutoff itself, we use the de Bruijn graph within Oases that is modified from Velvet’s original de Bruijn graph. For the other larger data sets, we compare the performance of our heuristic extension algorithm extABySS starting from the de Bruijn graph given by ABySS [10] against the performance of Trans-ABySS [13] that is a postprocessing module of ABySS. In each case, we compare the change recovered by Oases and Trans-ABySS to the change recovered by extVelvet and extABySS respectively over the values recovered by their base algorithms Velvet and ABySS respectively.

We applied each algorithm over *k*=25,31, and *c*=3,5,10 for smaller data sets and *c*=10,20,50 for larger data sets. BLAST is applied from predicted transcripts in Oases and Trans-ABySS, from paths in the de Bruijn graph in extVelvet and extABySS, and from contigs in Velvet/Oases and ABySS. When comparing each model organism against itself, nucleotide BLAST search is applied to a database of gene transcripts with initial *e*-value cutoff *e*_*i*_=10^−15^ and final *e*-value cutoff *e*_*f*_=10^−100^. In the other cases, translated BLAST search is applied to a database of protein transcripts in a related organism with initial *e*-value cutoff *e*_*i*_=10^−6^ and final *e*-value cutoff *e*_*f*_=10^−20^. For each transcript in the database, the top 8 nodes (and their twin nodes) are chosen to form the initial nodes for extension. Additional criteria are imposed to extend past very short nodes.

### Transcript recovery

**Fig. 3:**
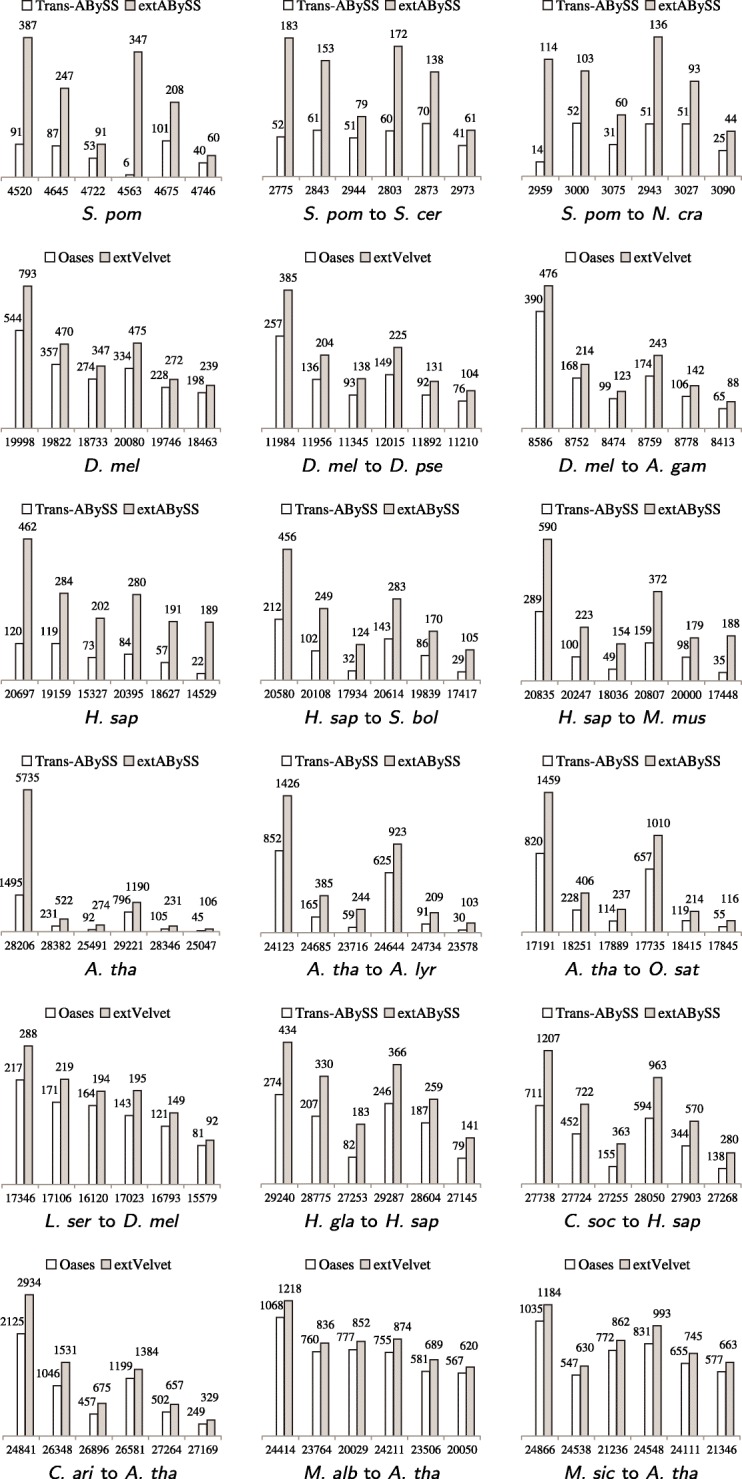
Comparisons of the change in the number of similar transcripts recovered by Oases and Trans-ABySS (shown as white bar) to the change in the number of similar transcripts recovered by extVelvet and extABySS (shown as grey bar) respectively over the number of similar transcripts recovered by Velvet and ABySS (shown under the *x*-axis) respectively for different values of *k* and *k*-mer coverage cutoff *c*. Within each graph, the corresponding values of *k*_*c* are 25_3, 25_5, 25_10, 31_3, 31_5, 31_10 from left to right for smaller data sets, including *D. melanogaster*, *L. sericata*, *C. arietinum*, *M. albus* and *M. siculus*, and 25_10, 25_20, 25_50, 31_10, 31_20, 31_50 from left to right for larger data sets, including *S. pombe*, *H. sapiens*, *A. thaliana*, *H. glaber* and *C. sociabilis*. When comparing each model organism against itself (graphs with a single-species label), nucleotide BLAST search is applied with *e*-value cutoff *e*_*f*_=10^−100^. In the other cases, translated BLAST search is applied with *e*-value cutoff *e*_*f*_=10^−20^

We assess the performance of each algorithm in recovering transcripts by investigating the amount of similar transcripts obtained and the amount of recovered transcripts that are close to full length. While the performance depends on the size of RNA-Seq data, the complexity of transcriptomes, the evolutionary distance between organisms and the assembly algorithm that is being used, Fig. 3 shows that Oases and Trans-ABySS recover more similar transcripts than their base algorithms Velvet and ABySS, while extVelvet and extABySS recover even more. The improvement of Trans-ABySS is small when compared to ABySS, which leads to a much larger improvement of extABySS over Trans-ABySS. These improvements are not absolute since different algorithms can recover different sets of similar transcripts.

**Fig. 4:**
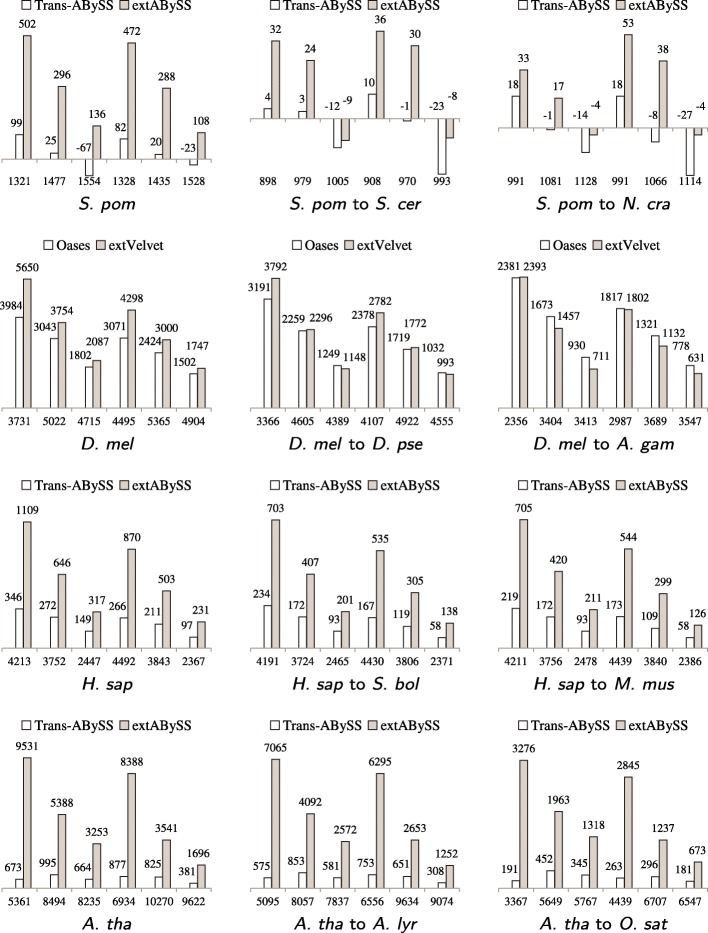
Comparisons of the change in the number of similar transcripts in the starting organism that are 80% full length transcripts (100% full length transcripts when *S. pombe* is the starting organism) and recovered by Oases and Trans-ABySS to the change in the ones recovered by extVelvet and extABySS respectively over the ones recovered by Velvet and ABySS respectively for different values of *k* and *k*-mer coverage cutoff *c*. Notations are the same as in Figure 3. These transcripts are the ones in which 80% (100% when *S. pombe* is the starting organism) of the coding region is included in the best BLAST alignment

Figure 4 shows that extVelvet and extABySS can recover more similar transcripts that are close to full length than Oases and Trans-ABySS in most cases. Both Oases and extVelvet (or Trans-ABySS and extABySS) can recover more full length transcripts than Velvet (or ABySS), which can be a few times more in some cases.

### Alternative splicing

**Fig. 5:**
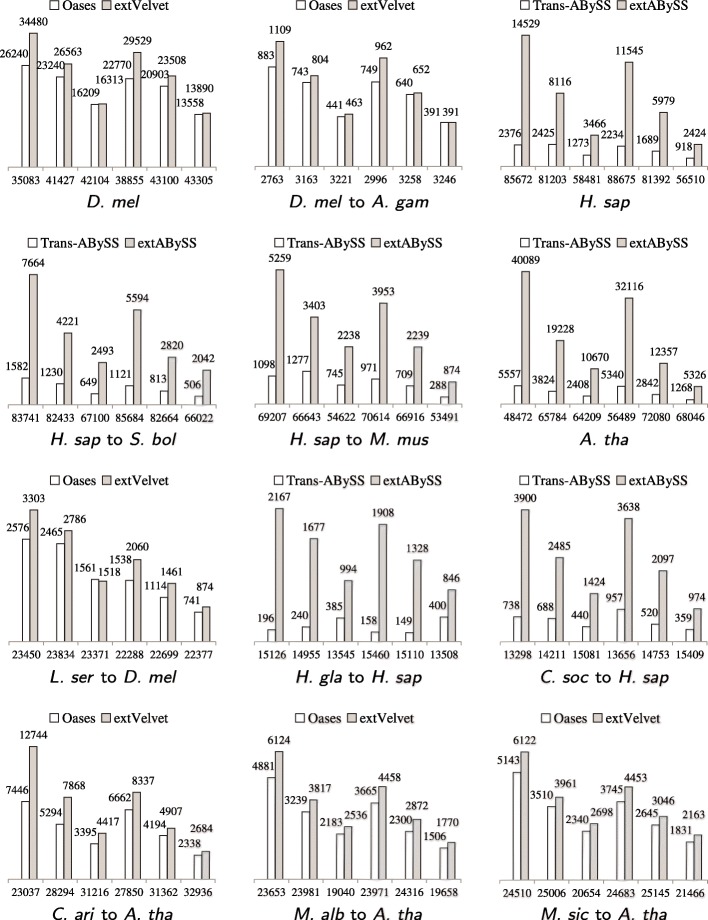
Comparisons of the change in the total number of exons in genes with multiple isoforms recovered by Oases and Trans-ABySS to the change in the ones recovered by extVelvet and extABySS respectively over the ones recovered by Velvet and ABySS respectively for different values of *k* and *k*-mer coverage cutoff *c*. Notations are the same as in Figure 3. Exons within isoforms that do not have the same starting position or the same ending position are considered to be distinct. An exon is recovered if it has some overlap with the best BLAST alignment. Exons within mRNAs are considered when comparing each model organism against itself, while exons within coding regions of the related model organism are considered in the other cases. Results for *S. pombe* are not included since there is little alternative splicing, while a few other results are not included due to poor annotations of alternative splicing in the related model organisms

We assess the ability of each algorithm in distinguishing between isoforms by considering exons in genes with multiple isoforms. Figure 5 shows that extVelvet and extABySS are able to recover a larger number of such exons in most cases.

**Fig. 6:**
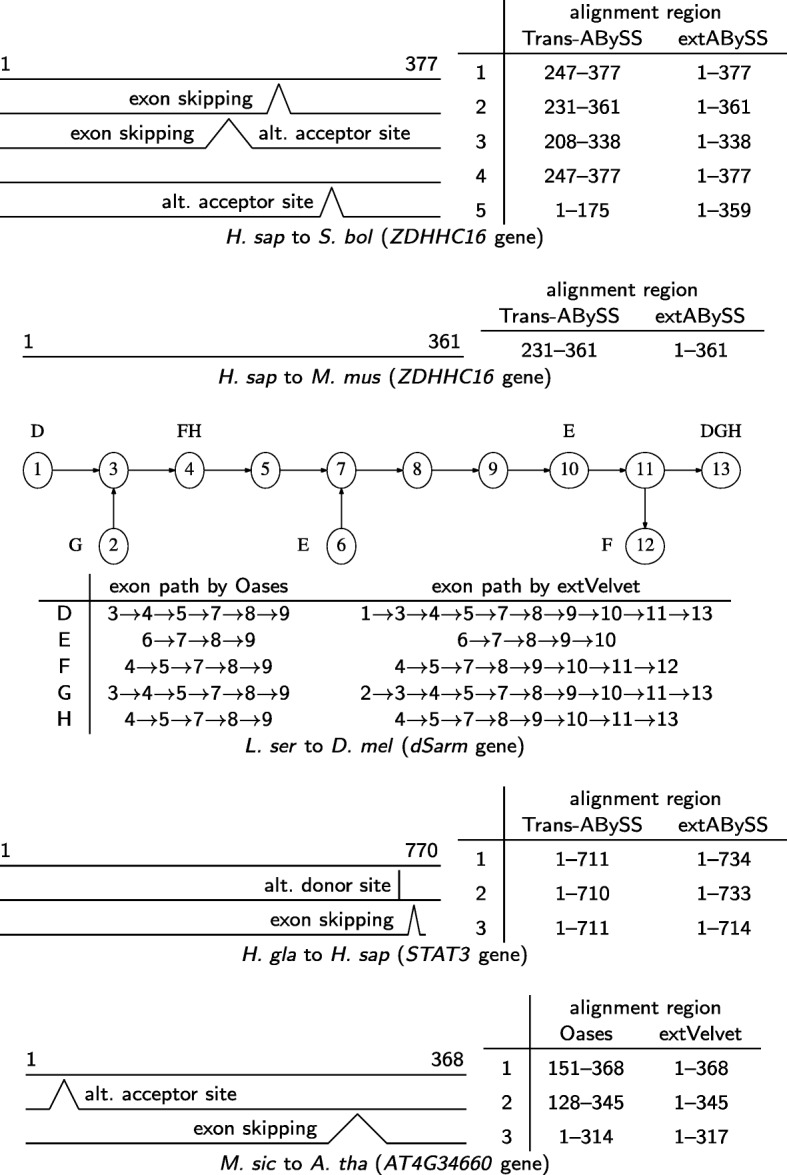
Examples of the resolution of alternative splicing with respect to a related organism. The splicing structures are on exons in the coding region of the related organism. For the *dSarm* gene, uppercase letters indicate isoforms and their start/end exons, with Oases resolving less isoforms than extVelvet. In the other splicing structures, the isoforms are drawn to scale and the starting and ending amino acid positions of isoform 1 are shown. For the *ZDHHC16* gene, Trans-ABySS cannot resolve its different isoforms on *S. boliviensis*, and recovers a shorter segment of it on *M. musculus* with no known alternative splicing. Trans-ABySS cannot resolve isoforms 1 and 3 of the *STAT3* gene, while Oases cannot resolve isoforms 1 and 2 of the *AT4G34660* gene

Figure 6 shows examples in which extVelvet and extABySS can better resolve isoforms with respect to a related organism, including the *ZDHHC16* gene, which is a zinc finger protein that may be involved in apoptosis regulation [29]; the *dSarm* gene, in which the loss of its function protects against injury-induced axon death [30]; the *STAT3* gene, which is an acute-phase response factor in which the isoforms have unique functions [31]; and the *AT4G34660* gene, which is a SH3 domain-containing protein that is involved in clathrin-mediated vesicle trafficking [32].

### Translocated transcripts

**Fig. 7:**
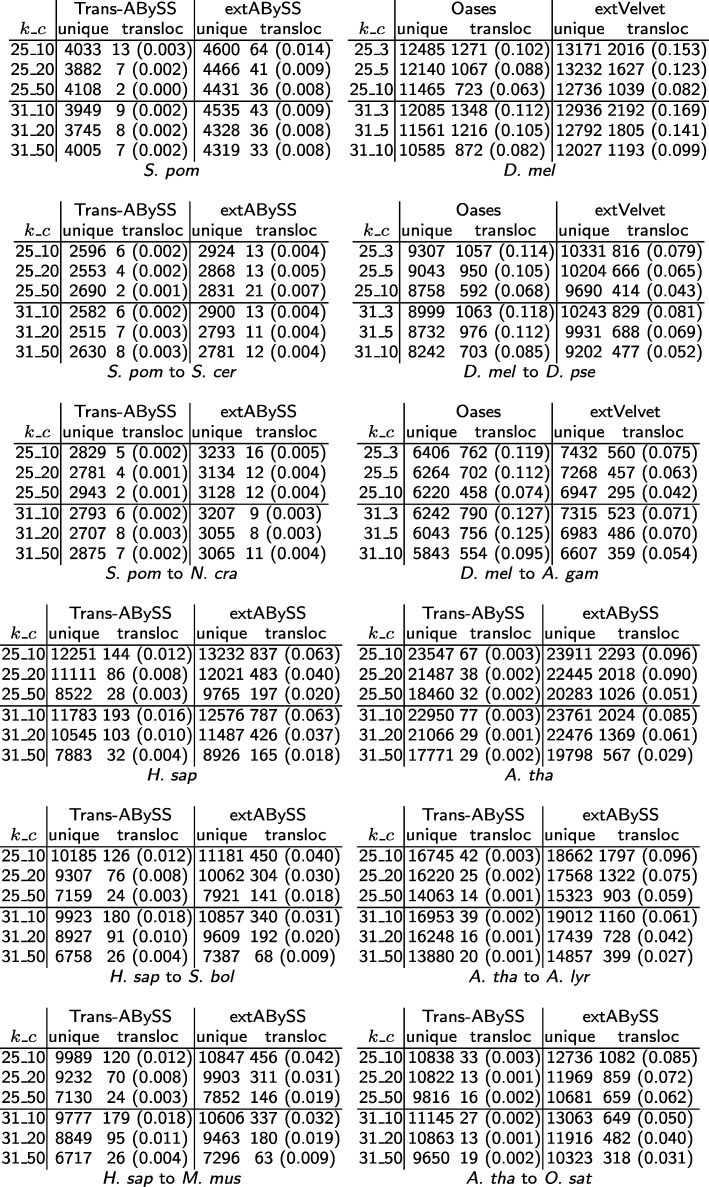
Comparisons of the number of similar transcripts in the starting organism that are uniquely mapped (unique) or translocated (transloc) as reported by GMAP and recovered by Oases and Trans-ABySS to the ones recovered by extVelvet and extABySS respectively for different values of *k* and *k*-mer coverage cutoff *c*. The number in parentheses is the ratio of the number of translocated transcripts to the number of uniquely mapped transcripts

We assess the reliability of each algorithm by identifying the amount of translocated transcripts that are returned. As reported by GMAP [33], Fig. 7 shows that extVelvet and extABySS recover a larger number of similar transcripts that are uniquely mapped than Oases and Trans-ABySS, with extVelvet returning less translocated transcripts than Oases when the starting organism is different from the related organism, and extABySS returning a few times more translocated transcripts than Trans-ABySS in most cases (except for *A. thaliana* when Trans-ABySS returns very few translocated transcripts).

### Gene expression

**Fig. 8:**
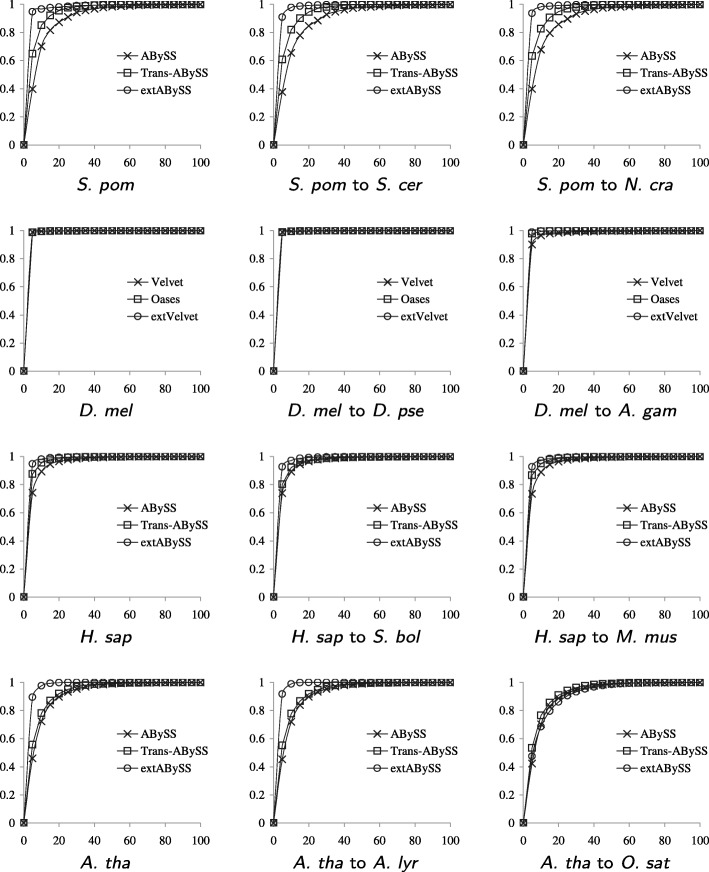
Comparisons of the cumulative distribution of the FPKM expression estimates of similar transcripts in the starting organism that are 80% full length transcripts (100% full length transcripts when *S. pombe* is the starting organism) and recovered by Velvet, Oases and extVelvet (or by ABySS, Trans-ABySS and extABySS), with the range of FPKM values in each assembly divided into 20 intervals of equal width and shown as a percentage under the *x*-axis. The least stringent values of *k*_*c* are used in each case, which is 25_3 for *D. melanogaster* and 25_10 for the other organisms

We assess the ability of each algorithm in recovering transcripts at different expression levels. We apply eXpress [4] to the reads in each data set with respect to the database of recovered similar transcripts in the starting organism that are close to full length to obtain FPKM expression estimates. Figure 8 shows that extABySS is able to recover a higher proportion of full length transcripts with low coverage than ABySS and Trans-ABySS.

### *Melilotus albus* and *Melilotus siculus*

**Fig. 9:**
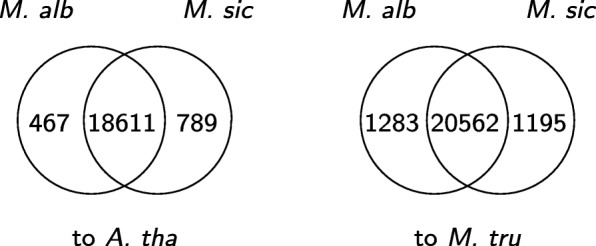
Venn diagrams of the number of genes that are present in recovered similar transcripts from *M. albus* and *M. siculus* when our algorithm is applied to *A. thaliana* and *M. truncatula* in the 25_3 assembly

**Fig. 10:**
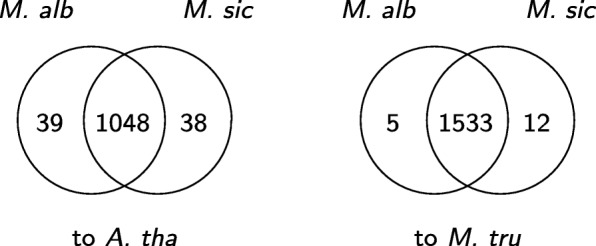
Venn diagrams of the number of significant GO terms from *M. albus* and *M. siculus* when our algorithm is applied to *A. thaliana* and *M. truncatula* in the 25_3 assembly

In order to study salt and waterlogging tolerance of the two *Melilotus* species, we apply our algorithm extVelvet starting from each species both to the model organism *A. thaliana* and to the non-model organism *Medicago truncatula*. Although *M. truncatula* is not as well annotated as *A. thaliana*, it is closer in evolutionary distance to *Melilotus* and will give better results. We assess the differences between the two species by applying GO Term Finder [34] to the two sets of genes that are present in recovered similar transcripts from *M. albus* and *M. siculus* when our algorithm is applied to *A. thaliana* and *M. truncatula*, and identify significant GO terms with Bonferroni corrected *p*-value below 0.01 within the biological process ontology. Figures 9 and 10 show that while a large number of genes in recovered similar transcripts and significant GO terms are shared by the two species, a small number of results that are unique to each species can be found (see Additional file 1 for details). For *M. albus*, the most notable unique genes are related to RNA splicing, response to brassinosteroid stimulus, and developmental regulation. For *M. siculus*, the most notable unique genes are related to response to karrikin (a smoke-derived molecule that regulates seed development), nucleic acid metabolism, negative regulation of cell differentiation, and nucleus organization. These results suggest large differences in gene expression strategies of these species, as they respond to the same stressful environments.

To assess gene expression under different conditions, we apply edgeR [35] on the FPKM expression estimates given by eXpress [4] to obtain a set of differentially expressed genes under one condition against another condition with *q*-value below 0.01, and apply GO Term Finder [34] to identify significant GO terms within each set of genes.

Tables 2 and 3 show that differentially expressed genes can be identified in all cases, with some of them associated with significant GO terms (see Additional file 1 for details). In the results from libraries associated with salt and waterlogging tolerance against control, many genes are found to be differentially expressed in *M. albus* that are related to response to chemical stimulus, stress, organic substance, inorganic substance, abiotic stimulus, and oxygen stress. There is also a significant enrichment of genes that respond to hormones, with at least one of these genes indicating ethylene physiology as important in the stress response. In contrast, very few genes are found to be differentially expressed in *M. siculus*. Among these genes, chalcone-flavanone, terpenoid, and ferulic acid physiology are implicated in the biology of the stress response. These results provide further basis to study the genes that are responsible for the major differences in salt and waterlogging tolerance of the two species.

**Table 2:** Number of differentially expressed genes recovered from *M. albus* and *M. siculus* when our algorithm is applied to *A. thaliana* and *M. truncatula* from libraries associated with one condition versus another condition in the 25_3 assembly, with organism indicating the starting organism and its related organism, SvsC indicating salt tolerance versus control, WvsC indicating waterlogging tolerance versus control, SWvsC indicating salt and waterlogging tolerance versus control, SWvsS indicating salt and waterlogging tolerance versus salt tolerance, and SWvsW indicating salt and waterlogging tolerance versus waterlogging tolerance

Organism	SvsC	WvsC	SWvsC	SWvsS	SWvsW
*M. alb* to *A. tha*	8	141	81	47	12
*M. sic* to *A. tha*	39	7	10	45	8
*M. alb* to *M. tru*	11	220	114	86	17
*M. sic* to *M. tru*	74	24	31	84	12

**Table 3:** Number of significant GO terms recovered from *M. albus* and *M. siculus* when our algorithm is applied to *A. thaliana* and *M. truncatula* from libraries associated with one condition versus another condition in the 25_3 assembly. Notations are the same as in Table 2

Organism	SvsC	WvsC	SWvsC	SWvsS	SWvsW
*M. alb* to *A. tha*	0	23	42	7	0
*M. sic* to *A. tha*	9	0	0	2	0
*M. alb* to *M. tru*	2	0	1	0	0
*M. sic* to *M. tru*	0	0	0	0	0

## Conclusions

Since the main memory requirement of our algorithm is for storing the de Bruijn graph and performing BLAST searches, our heuristic extension algorithms extVelvet and extABySS are much less memory intensive and more easily parallelizable than the base algorithms Velvet and ABySS [36]. Since a postprocessing module such as Oases may need more memory than its base algorithm Velvet, our heuristic extension algorithm provides an alternative in these cases. Iterative BLAST searches can be performed independently in parallel by assigning disjoint subsets of nodes to different processors.

The running time of our algorithm has large dependence on the number of nodes that are chosen for extension (see Table 4). This in turn depends on the size of RNA-Seq data and the complexity of transcriptomes, which are reflected by the number of nodes in the de Bruijn graph and the number of transcripts in the database. It also depends on the evolutionary distance between the starting organism and the related model organism. As the evolutionary distance increases, both the number of nodes that are chosen for extension and the running time decrease. When applying to a different related organism, our running time in terms of processor-hours is at most a few to 10 times more than the base algorithm in almost all cases, and it can be much less in some cases.

**Table 4:** Running time in processor-hours, with the values to the left and to the right of “+” indicating the running time of Velvet and Oases respectively (or ABySS and Trans-ABySS respectively), organism indicating the related model organism, time indicating the running time of extVelvet (or extABySS), chosen indicating the number of nodes that are chosen for extension, de Bruijn indicating the number of nodes in the de Bruijn graph, and database indicating the number of transcripts in the database

Least stringent *k*_*c*	Organism	Time	Chosen	De Bruijn	Database
*S. pom* (84+0.2)	*S. pom*	45	41692	536894	5011
	*S. cer*	12	15252	536894	5907
	*N. cra*	12	16366	536894	10082
*D. mel* (6.7+4.4)	*D. mel*	238	138972	459644	22102
	*D. pse*	67	64012	459644	16071
	*A. gam*	32	41580	459644	12659
*H. sap* (45+0.2)	*H. sap*	595	222244	1133368	32787
	*S. bol*	490	88340	1133368	25621
	*M. mus*	167	85166	1133368	29617
*A. tha* (112+0.2)	*A. tha*	2495	423410	3111862	41671
	*A. lyr*	944	218760	3111862	32549
	*O. sat*	616	144058	3111862	26777
*L. ser* (1.2+0.2)	*D. mel*	67	41872	257700	22102
*H. gla* (368+0.2)	*H. sap*	1920	192772	5457968	32799
*C. soc* (440+0.2)	*H. sap*	1344	175690	5030586	32799
*C. ari* (4.2+4.6)	*A. tha*	200	103524	1205362	41671
*M. alb* (5.8+2.9)	*A. tha*	79	82996	562210	41671
*M. sic* (9.3+6.8)	*A. tha*	67	83718	482826	41671

The situation is different in model organisms when similarity searches are performed to the organism itself. Since the BLAST hits are of much higher quality, path extensions can be very time-consuming. In such cases, mapping-first algorithms such as Cufflinks [2] or Scripture [1] could be used instead, which often have better performance since our need to impose a *k*-mer coverage cutoff to simplify the de Bruijn graph for heuristic extension often leads to missed transcripts.

Our heuristic extension strategy cannot be applied to all transcriptome assembly algorithms. On algorithms such as Trinity [12] that first clusters the data and constructs a de Bruijn graph individually for each cluster, each of these graphs has simple structures. Performing heuristic extension on top of these graphs will not lead to significant improvements.

While our strategy cannot replace transcript predictions in de novo assemblies when the goal is to identify novel transcripts that have no similarity to other organisms, we have shown that our strategy can recover more and longer transcripts and can better resolve isoforms when similar transcripts are available from a related organism. The sequence similarity support from the BLAST alignments ensures that the correspondences between the transcripts in the original organism and in the related organism are real.

## Additional file

Additional file 1 Lists of unique genes that are present in recovered similar transcripts and differentially expressed genes from libraries associated with one condition versus another condition along with significant GO terms recovered from *M. albus* and *M. siculus* when our algorithm is applied to *A. thaliana* and *M. truncatula* in the 25_3 assembly. (ZIP 101 kb)
